# Scoping review of the impact of coronavirus disease 2019 on unplanned pregnancy

**DOI:** 10.4102/phcfm.v14i1.3601

**Published:** 2022-12-15

**Authors:** Carmen S. Christian, Laura Rossouw

**Affiliations:** 1 Department of Economics, Faculty of Economic and Management Sciences, University of the Western Cape, Bellville, South Africa; 2 School of Economics and Finance, Faculty of Commerce, Law and Management, University of the Witwatersrand, Johannesburg, South Africa

**Keywords:** Sexual and reproductive health, lockdown, COVID-19, fertility, child-bearing intentions, contraceptive access

## Abstract

**Background:**

Increased pressure on the healthcare system because of coronavirus disease 2019 (COVID-19) along with national lockdown policies had consequences on the sexual and reproductive health of women. While the pandemic has resulted in changes in pregnancy intentions, child-bearing and fertility, the direction of this relationship is unclear and is likely to be impacted by each country’s socio-economic status and stage of fertility transition. Understanding the fertility trajectory and the pandemic is important in understanding population structures and ageing, which have consequences for health policies, budgeting and economic activity.

**Aim:**

This study aimed to conduct a scoping review of the impact of COVID-19 on unplanned pregnancy.

**Methods:**

A rapid review of available literature using Google Scholar, PubMed and Medical Literature Analysis and Retrieval System Online (MEDLINE), SocINDEX, Cumulative Index to Nursing and Allied Health Literature (CINAHL) Complete and Academic Search Ultimate. Articles in English from 2020 to 2021 were included.

**Results:**

Fifteen articles were included. These were mostly cross-sectional, primary data-collection surveys exploring the relationship between COVID-19 and child-bearing intentions.

**Conclusion:**

Access to contraceptives, socio-economic status and uncertainty about the health impact of COVID-19 on pregnancy were major themes that emerged when considering child-bearing intentions. Evidence of changes in the number of unplanned pregnancies and abortions was not insignificant but should be explored further. Although the studies covered a range of countries, more studies are needed focusing on low- and middle-income countries where the socio-economic impact of child-bearing intention is greater. There is a need for causal analysis using country-level data and for longer studies using more robust methodologies. The pandemic will continue to influence birth rates.

**Contribution:**

This article revealed gaps in the current literature on the measurement of the quantitative and causal impact of the COVID-19 pandemic on fertility and child-bearing. Findings from our study may assist in setting the trajectory for future research.

## Introduction

The onset of the coronavirus disease 2019 (COVID-19) pandemic in late 2019 changed various aspects of everyday existence and the way individuals interact with society. Coronavirus disease 2019 spread rapidly, and in lieu of an available vaccine, governments globally adopted lockdown policies to limit human contact and slow down viral transmission.^1^ Policies differed from country to country but included the closure of schools^2^ as well as nonessential services, encouraging social and public distancing and interruption of transport services.^3^

In addition to lockdown policies, public healthcare systems came under significant strain with the increase in the COVID-19 hospitalisations.^4,5^ Supply-chain disruptions further delayed the production and distribution of contraception.^6^ This increased pressure and the lockdown policies had consequences for the sexual and reproductive health (SRH) of women who had trouble accessing SRH services.^7^ Difficulty in accessing contraception and fertility treatments is likely to have consequences for human fertility and populations. Policies encouraging individuals to stay at home are also likely to have had an impact on fertility behaviours, especially coital frequency and the occurrence of unplanned pregnancies.^8^

Evidence from previous pandemics, such as the Ebola crisis in West Africa between 2013 and 2016, provides some insights into the impact of a global health crisis on SRH. In Liberia, Guinea and Sierra Leone, the distribution of family planning services declined by 65%, 51% and 23%, respectively, during the epidemic.^9,10^

While it is highly probable that the pandemic will result in a change in pregnancy intentions, child-bearing and overall fertility, the direction of this relationship is unclear. Aassve et al. theorise that the impact on overall fertility will differ by the country’s economic status and stage of fertility transition.^8^ In high-income countries, it is likely that the pandemic will result in a decline in pregnancy intentions and fertility, driven by disruptions in work–life balance, decreased access to assisted reproductive therapies for a relatively older population and large economic losses.

In low- and middle-income countries (LMICs), the impact of the pandemic on fertility is less clear. There is empirical evidence of increased fertility in LMICs during economic downturns, driven by the economic security that children are perceived to provide in the absence of credit markets and financial insurance. However, urbanisation of LMICs and the development of financial sectors means that children may no longer be a source of economic security, and family planning priorities may have shifted to smaller nuclear families. However, the shutdown of sexual and reproductive health services (SRHS) may have hampered access to contraception and abortion services, resulting in an increase in unplanned pregnancies.^8^

While the development of COVID-19 vaccines has created hope for returning to normal prepandemic life, the duration and globality of the crisis is likely to have had an impact on the fertility trajectory. Understanding the trajectory and the pandemic is key to understanding population structures and ageing, which in turn have consequences for health policies, health budgeting and economic activity. Evidence of the impact of the pandemic on unplanned pregnancies and subsequent fertility rates is limited, and the goal of this scoping review is to summarise the available evidence.

The aim was to conduct a scoping review of the impact of COVID-19 on unplanned pregnancy. More specifically, the review answered the following questions:

- What was the impact of the COVID-19 pandemic on child-bearing intentions?
- What are the factors that influenced unplanned pregnancies during COVID-19?
- What are the changes in the number of unplanned pregnancies and abortions because of COVID-19?
- What are the consequences of unplanned pregnancies during COVID-19?

## Methods

### Study design

This was a narrative scoping review of published literature to address these four research questions. The Preferred Reporting Items for Systematic Reviews and Meta-Analyses (PRISMA) reporting framework for scoping reviews was used as part of the methodology.

### Search strategy

A rapid review of the available literature was conducted using Google Scholar, PubMed or Medical Literature Analysis and Retrieval System Online (MEDLINE), SocINDEX, Cumulative Index to Nursing and Allied Health Literature (CINAHL) Complete and Academic Search Ultimate. The following limits were applied to all searches: English language, research published between 2020 and 2021. The search strings used a combination of Medical Subject Headings (MeSH) terms (‘COVID-19’, ‘pregnancy, unplanned’) and text words (‘unplanned pregnancy’, ‘child-bearing’, ‘child-bearing intentions’, ‘child* intentions’) used as indicated in Box 1. Given the recent context (COVID-19 pandemic), a cursory scan of the literature showed that there was a limited number of original research on this topic published in peer-reviewed journals. For this reason, it was decided to include original research published in less rigorously reviewed formats as well (e.g. preprints, working papers, etc.). Our main goal was to identify articles that could shed light on our review questions.

BOX 1 Search strings used in scoping review.

**Search strings:**

‘COVID-19’ and ‘pregnancy, unplanned’

‘COVID-19’ and ‘unplanned pregnancy’

‘COVID-19’ and ‘child-bearing’

‘COVID-19’ and ‘child-bearing intentions’

‘COVID-19’ and ‘child† intentions’

COVID-19, coronavirus disease 2019.

†, wild card (used to take the place of one or more characters in a search term when searching).

The list of articles found during the search was combined and duplicates were removed. Relevant articles were then screened by title and abstract. At each stage in the search process, the number of included and excluded studies was recorded as well as the reasons for exclusion. The final list of articles was screened by obtaining the full text, and reasons for further exclusions were recorded.

The researchers performed the search and screening process in tandem. Uncertainty about including or excluding articles were resolved by discussion. The reference lists of included full-text articles were examined for additional eligible literature.

### Extraction of data

The following data were extracted into a standardised template:

1. Authors
2. Year of publication
3. Location of the study population
4. Aim or purpose of the publication
5. Type of publication
6. Methods (if relevant, a summary of the methods used)
7. Study limitations as reported by the authors
8. Description of the impact of COVID-19 on child-bearing intentions
9. Evidence on the factors that influenced unplanned pregnancies
10. Evidence on the changes in the number of unplanned pregnancies (and abortions)
11. Evidence on the consequences of unplanned pregnancies

Data for extraction points (1) to (7) are presented in Table 1 in the review findings, while data for extraction points (8) to (11) are presented in a narrative format only.

**TABLE 1:** Studies included in the scoping review.

Article	First author	Year	Location	Aim or purpose of publication	Publication type	Study design	Limitations
1.	Afolabi et al.^11^†	2021	Nigeria	To examine the implications of the COVID-19 lockdown on fertility, economic and intimate partner violence in Nigeria	Original research (preprint)	Cross-sectional, qualitative, descriptive, analytical	Possible selection bias, and cross-sectional design cannot infer causality
2.	Hunie Asratie et al.^12^	2021	Ethiopia	To assess unintended pregnancy during the COVID-19 pandemic and its associated factors among women attending antenatal care in northwest Ethiopia	Original research	Cross-sectional, descriptive, analytical	None reported
3.	Berrington et al.^13^	2021	United Kingdom (UK)	To examine the recent declines in period fertility in the constituent countries of the UK during the past decade and speculate on the mechanisms through which the COVID-19 pandemic could influence child-bearing intention in the UK	Working article	Observational, analytical	None reported
4.	Coombe et al.^14^	2021	Australia	To investigate the impact COVID-19 lockdown regulations had on sexual and reproductive health (SRH)	Original research	Cross-sectional, qualitative, descriptive, analytical	Selection bias and convenience sampling limit inference to general population
5.	Flynn et al.^15^	2021	United Kingdom, South Africa, Nigeria, United States, Republic of Ireland, Kenya, Australia, India, Ghana, Canada, Pakistan, Uganda, United Arab Emirates, Zambia, Honduras, Indonesia, Jamaica, Kazakhstan, Russian Federation, South Sudan, Switzerland, Trinidad and Tobago, Azerbaijan, Barbados, Germany, Greece, Guyana, Italy, Malawi, Namibia, the Netherlands, New Zealand, Saint Vincent and the Grenadines, Thailand and Zimbabwe	To investigate how the COVID-19 pandemic influenced pregnancy-planning behaviours	Original research	Cross-sectional qualitative, descriptive	Social, environmental and psychological temporal changes were not tracked. Possible social desirability bias or biases in the recruitment method
6.	Lewis et al.^16^	2021	Scotland	To explore young people’s experiences of accessing and using condoms and contraception in the early months of the COVID-19 pandemic and its implications	Original research	Cross-sectional, qualitative	Cross-sectional design precludes assessment of change over time
7.	Lin et al.^17^	2021	United States of America	To assess the impact of the COVID-19 pandemic on economic conditions and reproductive health decisions related to child-bearing and pregnancy; specifically, this study evaluates if, during the initial months of the COVID-19 pandemic, vulnerable populations experience different financial and reproductive health outcomes compared to the general population	Original research	Cross-sectional, descriptive, analytical	Recall bias, selection bias and social desirability bias
8.	Suzuki et al.^18^	2021	Japan	To examine the frequency, associated factors and outcomes of specific expectant mothers managed at the Japanese Red Cross Katsushika Maternity Hospital under the COVID-19 epidemic compared with those in 2019 as reported previously	Short communication (of original research)	Comparative retrospective analysis	None reported
9.	Caruso et al.^19^	2020	Italy	To investigate the effects of social distancing during the COVID-19 pandemic on the use of hormonal contraceptives, their discontinuation and the risk of unplanned pregnancy	Clinical study	Observational, cross-sectional descriptive	Did not include information programme to educate participants
10.	Haddad et al.^20^	2020	Lebanon	To evaluate the socio-economic and psychological factors related to current pregnancy status and unwanted pregnancy among a sample of Lebanese women during the COVID-19 lockdown	Original research (preprint)	Cross-sectional, descriptive, analytical	Cross-sectional design cannot infer causality; potential selection bias, information bias, residual confounding bias
11.	Lindberg et al.^21^	2020	United States of America	To examine how cisgender women in the United States feel that the COVID-19 pandemic has influenced their sexual reproductive health and to examine women’s reports of pandemic-related economic challenges and how these challenges intersect with their sexual and reproductive experiences	Survey report	Cross-sectional, descriptive	None reported
12.	Luppi et al.^22^	2020	Italy, Germany, France, Spain and the United Kingdom	To describe changes in young people’s fertility plans – that is, in couples’ intention to have a child soon – because of the COVID-19 crisis at the start of the health emergency in Europe	Original research	Cross-sectional, retrospective, descriptive, analytical	Recall bias
13.	Riley et al.^23^	2020	132 LMICs in Africa, Asia, Eastern and Southern Europe, Latin America and the Caribbean	To analyse what is at stake if government actions and provision of resources during the COVID-19 pandemic do not ensure that essential sexual and reproductive health services continue	Comment (of original research)	Observational, cross-sectional, analytical	None reported
14.	Wilde et al.^24^	2020	United States of America	To use Google Trends data to predict the effect of the COVID-19 pandemic on future births in the United States	Discussion article	Observational analytical	None reported
15.	Zhu et al.^25^	2020	China (Shanghai)	To evaluate fertility intentions among couples in Shanghai under the novel coronavirus infection (COVID-19) pandemic against the backdrop of persistently low fertility	Clinical article	Cross-sectional (single-centre), descriptive, analytical	Possible ‘Berkson’s bias’, small sample size, cross-sectional survey focused on intention (as opposed to behaviour)

Note: Please see full reference list of the article, Christian CS, Rossouw L. Scoping review of the impact of coronavirus disease 2019 on unplanned pregnancy. Afr J Prm Health Care Fam Med. 2022;14(1), a3601. https://doi.org/10.4102/phcfm.v14i1.3601

COVID-19, coronavirus disease 2019; LMICs, low- and middle-income countries.

† , It appears that the authors made a calculation error in this article by using the incorrect denominator. We amended the statistic by using the denominator that was used consistently in the article, that is, 245.

### Data analysis

Data were analysed qualitatively to compare different methodologies and identify knowledge gaps based on our extraction template. An inductive approach was used to analyse themes. We did not conduct a critical appraisal given that it is not a prerequisite of a scoping review, nor necessary for our research agenda (to review rapidly emerging evidence on this topic before embarking on original research). However, we extracted data on the limitations reported in each study (where reported) and based on it, we sensitise readers to the weaknesses of some study designs. Where available, confidence intervals are included in the given write-up. If not shown, the article reviewed did not provide confidence intervals.

## Review findings

**FIGURE 1:**
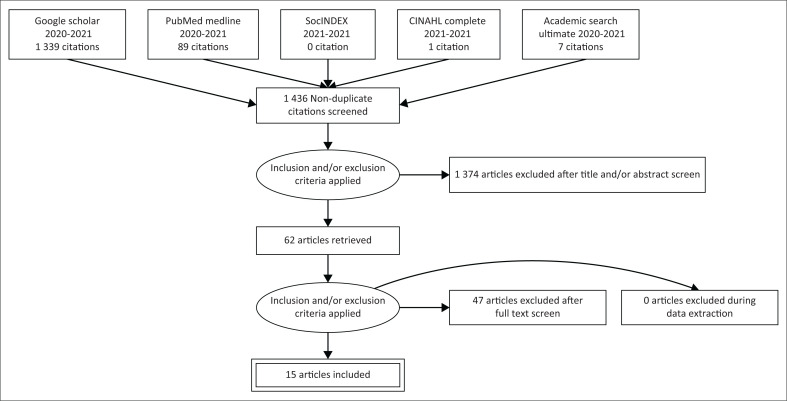
Flow diagram for selection of articles.

The search strategy resulted in 1489 articles (Figure 1). Once duplicates (53) were removed, 1436 full-text articles remained. These were assessed and an additional 1374 were excluded after screening the title and abstract, leaving 62 publications for further screening. Forty-seven articles were excluded after the full-text screen, leaving a total of 15 articles.

### Features of the included articles

In total, 15 published articles were included in the review (Table 1). All of these were published in 2020 or the first half of 2021, and the majority (9 out of 15) focused on developing countries (Table 2).

**TABLE 2:** Features of included articles.

Characteristic	*n*
**Year of publication**	
2021	8
2020	7
**Location of publication**	
Single developing country only	4
Single developed country only	8
Multiple developing countries	1
Multiple developed countries	1
Mixed (developed and developing countries)	1
**Publication type**	
Original research	7
Preprint	1
Working or discussion article	2
Short communication or comment	2
Survey report	1
Clinical study or article	2
**Study design**	
Cross-sectional qualitative descriptive analytical	2
Cross-sectional qualitative descriptive	1
Cross-sectional qualitative	1
Cross-sectional descriptive analytical	3
Cross-sectional descriptive	1
Cross-sectional retrospective descriptive analytical	3
Comparative retrospective analysis	1
Observational cross-sectional descriptive	1
Observational analytical	2

Most of the articles reviewed were original research (7 out of 15). In terms of study design, the most prevalent type was cross-sectional that relied on surveys or secondary data (12 out of 15). No studies using randomised-control trials or panel data analysis were found at the time of searching. Six of the articles (6 out of 15) did not report on study limitations. Only four articles (4 out of 15) reported their cross-sectional design as a study limitation, even though most studies were cross-sectional (12 out of 15). Different biases were frequently reported as limitations: ‘Berkson’s bias’ (1 out of 15), recall bias (2 out of 15), selection bias (4 out of 15), social desirability bias (2 out of 15), information bias (1 out of 15) and residual confounding bias (1 out of 15). One study (1 out of 15) reported its small sample size as a limitation, while another (1 out of 15) reported its lack of an information programme to educate participants as an oversight.

### Description of findings

#### Impact of coronavirus disease 2019 on child-bearing intentions

Most of the articles reviewed did not measure the impact of COVID-19 on child-bearing intentions directly (no causal studies) but instead measured the prevalence and magnitude of child-bearing intentions during the pandemic period. As such, most of the evidence presented is descriptive statistics. Some articles do not provide descriptive statistics but provide comments on their views of the impact of the pandemic on reproductive health experiences: ‘The economic and social instability of the pandemic is likely to contribute to ongoing declines in child-bearing in the United States’.^21^

Table 3 summarises the evidence of reported levels of child-bearing intentions during COVID-19. Only one of the articles under review suggests a predominantly positive relationship between COVID-19 and child-bearing intentions. In this article, respondents were asked to self-report an increase or decrease in their child-bearing intentions. Forty-one per cent of respondents self-reported an increase in child-bearing intentions over the period. Instead, most of the evidence supports a negative relationship between COVID-19 and child-bearing intentions (up to 72% of respondents) or no or unclear changes in intentions.

**TABLE 3:** Descriptive statistics on child-bearing intentions during coronavirus disease 2019 (2020–2021).

Impact	Article	Description
Mainly increased intentions	Lin et al.^17^	When asked about how their desire to be pregnant has changed during the pandemic, 41% reported an increase in child-bearing intentions, 25% reported a decrease in child-bearing intentions and 34% reported no change in their intentions.
Mainly decreased intentions	Flynn^15^	The COVID-19 affected pregnancy planning differently, but mostly delayed pregnancy intention. Twenty-seven per cent of respondents reported that they brought their pregnancy plans forward, while 71.9% reported that they planned to delay their pregnancy plans.
	Wilde^24^	The article predicts that there will be a massive decrease in fertility during the pandemic. The findings point to the trend in overall fertility, rather than child-bearing intentions per se.
	Lindberg^21^	The COVID-19 affected pregnancy planning differently, but mostly delayed pregnancy intention. Thirty-four per cent of women interviewed reported that they would delay their pregnancies or decrease their number of pregnancies because of the pandemic. Seventeen per cent of women wanted to have children sooner and have more children because of the pandemic.
No change or unclear	Coombe^14^	Most participants reported no impact on their future plans for pregnancy (reporting variations of ‘no’ or ‘no impact’). However, some participants reported delaying or avoiding pregnancy because of the pandemic.
	Luppi et al.^22^	The authors found varying results across countries. During the COVID-19 pandemic, the majority of respondents planned to postpone their child-bearing intentions, as opposed to abandoning it completely. The authors differentiate between individuals whose child-bearing intentions were unchanged (Italy = 25.56%, Germany = 30.07%, France = 32.03%, Spain = 21.17%, UK = 23.04%), postponed (Italy = 37.93%, Germany = 55.1%, France = 50.70%, Spain = 49.57%, UK = 57.78%) and child-bearing intention abandoners (Italy = 36.51%, Germany = 14.02%, France = 17.27%, Spain = 29.26%, UK = 19.18%). The prevalence of abandoners in Italy was substantially higher than in other countries.
	Zhu et al.^25^	Child-bearing intentions remained relatively unchanged for 66.02% of participants; 33.08% of participants reported that their child-bearing intentions were affected by the pandemic.

Note: Please see full reference list of the article, Christian CS, Rossouw L. Scoping review of the impact of coronavirus disease 2019 on unplanned pregnancy. Afr J Prm Health Care Fam Med. 2022;14(1), a3601. https://doi.org/10.4102/phcfm.v14i1.3601

COVID-19, coronavirus disease 2019; UK, United Kingdom.

#### Evidence of changes in the magnitude and prevalence of unplanned pregnancies and abortions during COVID-19

Findings from the studies reviewed with evidence of changes in the reported number of unplanned pregnancies and abortions during COVID-19 were not insignificant and varied from 10% to 47%, depending on the sample characteristics.

In a Nigerian study, there was a general perception amongst respondents (155 [63.3%] men and 90 [36.7%] women) that lockdown led to an increase in unintended pregnancies (mean = 3.90 and s.d. = 0.93 from five Likert scales).^11^ In this study, the demographic indicators show that of the total of 245 respondents, 103 (42%) reported that they did not want another child, while 78 (31%) said they wanted another child and 57 (23%) reported that they might consider it later. But 10% (24 out of 245) said the lockdown contributed to their pregnancy while 78% (193 out of 245) said it did not. The study also found that more than 48% (119 out of 245) said they were using contraceptives to prevent pregnancy while 40% (98 out of 245) said they were not, and 4.5% (11 out of 245) said they might consider using contraceptives at a later stage.

Another study found that in a group of women from Italy who had not abided by social distancing and continued their sexual activity despite discontinued short-acting reversible contraceptive (SARC) use, 31.9% (15 out of 47) had an unplanned pregnancy and sought an abortion.^19^ A study from Japan focused on pregnant women with social problems, also called ‘specific expectant mothers’ (i.e. pregnant women with at least one social risk factor) and found that 16.8% (73 out of 1650) and 13.8% (31 out of 171) had unplanned pregnancies in 2019 and 2020.^18^

At the upper end of the spectrum, the magnitude of unintended pregnancy was 47.17% (95% confidence interval [CI] 42.2% – 52.2%) among Ethiopian women attending antenatal care during the COVID-19 pandemic.^12^ In Lebanon, a study found that 22.0% of women who were pregnant reported their pregnancies as unwanted.^20^ More specifically, a significantly higher proportion of unwanted pregnancies was found among women who did not regularly visit their physician (57.1%) and those with a history of unwanted pregnancy (80.0%), while in contrast, women who visited their physician for routine check-ups had a lower probability of unwanted pregnancy.

A study that focused on 132 LMICs estimated that a proportional decline of 10% in the use of short- and long-acting reversible contraceptives because of reduced access as a result of COVID-19 lockdown would result in an additional 15 million unintended pregnancies over the course of a year.^23^

The same study also considered the potential *consequences* on health outcomes of countrywide lockdown that directly impacted SRHS.^23^ Under lockdown, SRHS would be affected either through forced clinic closures or recategorisation of abortions as nonessential services. They estimated that if 10% of women who under pre-COVID-19 conditions would have had a safe abortion resorted to unsafe methods instead, an additional 3.3 m unsafe abortions would take place in LMICs over a year. In turn, this increase in unsafe abortions would lead to an additional 1000 maternal deaths over the same period.

#### Factors associated with child-bearing intentions during coronavirus disease 2019

Some studies went beyond reporting the magnitude of child-bearing intentions during COVID-19 and provided some evidence of factors related to child-bearing intentions during the period under review. Some pertinent factors include access to contraception, socio-economic status and health status.

**Access to contraception:** The pandemic acted as a barrier to accessing various SRHS. One study found that 20% of their sample of contraception users reported an increased difficulty in accessing contraception during the pandemic. This was largely because of an inability to access prescriptions and pharmacies, although some respondents cited affordability concerns.^17^ Similarly, another study found that 16.1% of the women in their sample decreased their use of SARC during the pandemic.^19^ Of those who discontinued SARC, 92.1% continued their sexual activity. At the higher end of the spectrum, one study reported that 59.8% of their sample disclosed that the social-distancing policies related to country lockdown policies limited their access to contraception,^16^ while in another study, 33% of their sample either had to delay or cancel sexual and reproductive healthcare because of the pandemic.^21^

While the pandemic limited access to SARC, it also resulted in limited services to have long-acting reversible contraception (LARC) removed. Among respondents who postponed getting pregnant during the pandemic, 20% reported being unable to access services for the removal of their contraceptive device,^15^ while others reported incidents of difficulty getting LARC inserted or removed.^17^

The impact of even a small reduction in access to contraception will have affected many. Evidence of this is found in a study that estimated that a 10% proportional reduction in all forms of contraceptives in LMICs would have resulted in 49 m women having an unmet need.^23^

**Socio-economic status:** Affordability concerns about pregnancies and future earnings potential had a bearing on fertility plans, and this seems to have disproportionately affected women from lower socio-economic status backgrounds. Thirty-seven per cent of lower-income women in the United States postponed their pregnancy during the pandemic, compared with 32% of higher-income women.^21^ Similarly, evidence from respondents in the United States who reported an inability to afford food, transport and housing showed that they had twice the odds of a decreased desire to become pregnant during the pandemic compared with respondents who could afford these necessities.^17^ In addition, two other studies reported that concerns about future employment and income security were considered when postponing pregnancies during the pandemic.^15,22^

The role that socio-economic status plays in influencing fertility intentions during the pandemic is also evident when we observe the level of education. However, the direction of the relationship is less consistent. In line with the expected direction, evidence from the United Kingdom and Italy shows that having a tertiary degree results in respondents not altering their child-bearing plans.^22^ However, in Germany, France and Spain, tertiary education was associated with abandoning or postponing child-bearing plans until after the pandemic.^22^ Among a sample of women in China, women with lower levels of education were more likely to have changed their pregnancy plans because of the pandemic, although it is unclear in what manner the plans were changed.^25^

**Health status:** The uncertainty around the health impact of COVID-19 on pregnancy outcomes also factored into women’s child-bearing decisions. A multicountry study found that 52.6% of respondents who postponed their pregnancy during the pandemic did so because of concerns about the impact of the virus on the health of the foetus.^15^ Similarly, a study in China found that 62.5% of respondents who cancelled their pregnancy were concerned about foetal health.^25^ Likewise, participants in an Australian study cited concerns about pregnancy care during the pandemic, not putting undue strain on the healthcare system and concerns about the impact of the virus on pregnant women and newborns.^14^

In addition, postponing pregnancies was associated with an expected lack of pregnancy care during the pandemic as healthcare services were redirected.^15^

Women’s history of fertility and gynaecological health also had an impact on their pandemic child-bearing intentions. Women with a history of gynaecological diseases were less likely to change their pregnancy plans during the pandemic, compared with those with no history of gynaecological diseases.^25^

**Other factors:** Another minor factor related to child-bearing intentions during the pandemic includes age. Women aged 24 or older were less likely to alter their child-bearing intentions or fertility plans compared with their younger counterparts.^22^

Sexual orientation and population group also played a role in child-bearing intention during the pandemic.^21^ More specifically, in the United States, black people (44%) and Hispanic women (48%) were more likely than white (28%) women to report that they were postponing their pregnancy or wanted fewer children because of the pandemic. The same was true for queer (46%) compared with straight (33%) women.

In China, support for government lockdown policies was also associated with a lower likelihood of cancelling pregnancy plans during COVID-19.^25^

Participants in a study in Australia reported putting plans to conceive on hold either because of the cancellation of IVF services or because of the pandemic.^14^

## Implications and recommendations

In this article, we reviewed the available evidence on the impact of the COVID-19 pandemic on fertility and child-bearing intentions. Most studies analysed in this article are cross-sectional, primary data–collection surveys exploring the relationship between COVID-19 and child-bearing intentions. Although the studies reviewed covered a range of countries, we recommend that more studies focus on LMIC populations. Our review found that in several studies, socio-economic status was associated with fertility intentions, with affordability concerns playing a role. Given this, one might expect the response in a LMIC context to be different than in an HIC context.

The review highlights the absence of causal analysis using country-level data and the quantification of the impact of the pandemic on fertility rates. We therefore recommend that more causal studies be conducted so that the impact of COVID-19 can be clearly identified.

Access to contraceptives, socio-economic and health status are major themes that emerge when considering the correlates of child-bearing intentions during COVID-19. These should be further explored using more robust methodologies over longer time periods to infer causality. Likewise, evidence of changes in the number of unplanned pregnancies and abortions during COVID-19 were not insignificant, but we recommend that these be further explored given the relatively small sample sizes and methodologies used in studies.

We acknowledge that the period of analysis (2020 to June 2021) is too short to capture the impact of lockdown policies on fertility adequately. The pandemic is likely to influence the annual number of live births for the coming years. Studies on demographic changes after natural disasters have found that the immediate and medium-term effects on fertility often differ.^8^
